# Functional Analysis of the Molecular Interactions of TATA Box-Containing Genes and Essential Genes

**DOI:** 10.1371/journal.pone.0120848

**Published:** 2015-03-19

**Authors:** Sang-Hun Bae, Hyun Wook Han, Jisook Moon

**Affiliations:** 1 College of Life Science, Department of Bioengineering, CHA University, Seoul, Korea; 2 General Research Institute, Gangnam CHA General Hospital, Seoul, Korea; Univeristy of California Riverside, UNITED STATES

## Abstract

Genes can be divided into TATA-containing genes and TATA-less genes according to the presence of TATA box elements at promoter regions. TATA-containing genes tend to be stress-responsive, whereas many TATA-less genes are known to be related to cell growth or “housekeeping” functions. In a previous study, we demonstrated that there are striking differences among four gene sets defined by the presence of TATA box (TATA-containing) and essentiality (TATA-less) with respect to number of associated transcription factors, amino acid usage, and functional annotation. Extending this research in yeast, we identified KEGG (Kyoto Encyclopedia of Genes and Genomes) pathways that are statistically enriched in TATA-containing or TATA-less genes and evaluated the possibility that the enriched pathways are related to stress or growth as reflected by the individual functions of the genes involved. According to their enrichment for either of these two gene sets, we sorted KEGG pathways into TATA-containing-gene-enriched pathways (TEPs) and essential-gene-enriched pathways (EEPs). As expected, genes in TEPs and EEPs exhibited opposite results in terms of functional category, transcriptional regulation, codon adaptation index, and network properties, suggesting the possibility that the bipolar patterns in these pathways also contribute to the regulation of the stress response and to cell survival. Our findings provide the novel insight that significant enrichment of TATA-binding or TATA-less genes defines pathways as stress-responsive or growth-related.

## Introduction

To adapt to rapidly changing environments, cells often regulate gene expression at the transcriptional level. At this level, TATA box elements influence the recruitment of cofactors and transcription factors to the promoter regions of target genes. The promoters of genes containing TATA boxes are occupied by regulatory molecules that differ from those that bind to the promoters of TATA-less genes, greatly contributing to the differences in response between these two gene sets: TATA-containing genes tend to participate in responses that are necessary for stress-related gene expression, whereas TATA-less genes are associated with cell growth. This indicates that differences in sequence-specific elements in the promoter region could confer distinct functional effects on cells [1].

Because TATA-containing genes are extensively regulated, their regulation is suitable for the mounting of stress responses or stochastic responses by the cell. Growth-related genes tend to be TATA-less, to display robust expression and to be expressed in a growth-related manner [2–4]. Thus, switching between mutually exclusive gene regulatory events is important for balancing growth and stress responses within the organism [5].

Our previous study revealed that there are striking differences among four gene sets defined by the presence of a TATA box (TATA-containing) and essentiality (TATA-less) with respect to number of transcription factors, amino acid usage, and functional annotation [6]. Extending this research in yeast, the aim of the present study is to determine whether enrichment of TATA-containing or TATA-less genes classifies KEGG pathways [7] into functionally relevant groups and, if so, whether such groups demonstrate certain patterns of gene expression, based on the view that molecules interact with each other by forming complexes or by being expressed in a sequential manner and thereby contributing to cellular responses. To the best of our knowledge, yeast KEGG pathways have not been clustered and analyzed according to whether TATA-containing or TATA-less genes are enriched in the maps of these pathways. We defined TATA-containing-gene-enriched pathways as TEPs and essential-gene-enriched pathways as EEPs, two categories that did not overlap.

Our study demonstrated that growth-related pathways such as transcription and translation are overrepresented for EEPs, whereas TEPs are biased toward metabolism, especially carbohydrate metabolism. Furthermore, TEPs and EEPs show opposite patterns with respect to functional category, transcriptional regulation, codon adaptation index and network properties. These results provide new insight into how the enrichment of TATA-containing or TATA-less (‘essential’) genes determines the properties of molecular interactions.

## Materials and Methods

### Yeast coding gene data

The yeast ORF (open reading frame) information (5,649) was downloaded in a tab-separated form from SGD (Saccharomyces Genome Database, http://www.yeastgenome.org/).

### Essentiality and TATA box information

Gene lethality (1,101) and TATA (1,089) box information was obtained from MIPS (http://mips.helmholtz-muenchen.de) and from supplementary data in the published literature (2).

### KEGG pathways

The yeast pathway data were collected from KEGG API (application programming interface). KEGG enzyme information was obtained from http://rest.kegg.jp/link/enzyme/sce. The enzymes were classified according to the enzyme commission number, which consists of four numbers separated by periods (e.g., EC 1.9.3.1). For efficient integration of the heterogeneous data, all of the Entrez Gene IDs provided by KEGG were converted to the ORF names. Diagrams of metabolic map, ribosome biogenesis and glycolysis were obtained using Pathview, R package [8].

### Transcription complex information

Information on the preferential use of SAGA and TFIID was obtained from the supplementary data in the published literature [3].

### Transcription factor information

The documented and expected transcription factor data were obtained in HTML format from Yeastract (http://www.yeastract.com/) and processed to obtain the information in a tab-separated format. The number of transcription factors linked to each gene was counted, and the average for each pathway was calculated.

### Molecular interaction degree

Yeast interaction data (5980 nodes, 322630 edges) was extracted in a tab-separated form from SGD (Saccharomyces Genome Database, http://www.yeastgenome.org/).

### Finding highly connected molecular complexes using MCODE (molecular complex detection)

Yeast molecular interaction data (http://www.yeastgenome.org/) was subject to analysis for finding highly connected networks via the Cytoscape package, MCODE [9].

### Visualization

All of the graphs presented in this work were created using the R graph packages and Cytoscape [8, 10, 11].

### Statistical analysis

The analysis of the enrichment of gene sets was conducted through a hypergeometric distribution [12]. For the correction of multiple comparisons, the p-values were adjusted using the Benjamini–Hochberg procedure (BH-step-up procedure) or the Bonferroni correction (in the case of enzyme enrichment, i.e., few comparisons). The Shapiro test was used to test the normality of the data, and the F-test was used to compare the variance of datasets consisting of more than two samples. All of the statistical analyses were performed using the R program.

## Results

### TATA-containing-gene-enriched pathways did not overlap with essential-gene-enriched pathways

Essential genes tend to be TATA-less; therefore, we define TATA-less genes as essential genes hereafter in this work. Initially, we separated the KEGG pathways into two groups: TATA-containing-gene-enriched pathways (TEPs) and essential-gene-enriched pathways (EEPs). Using hypergeometric distribution, the significance of enrichment was determined using as a criterion an adjusted p value of less than 0.05. Of 102 KEGG pathways, TEPs comprised 25 pathways and EEPs 23; the remaining 54 pathways were not associated with either of these groups. In our study, we focused only on genes classified with the groups TEPs and EEPs.

**Fig 1:**
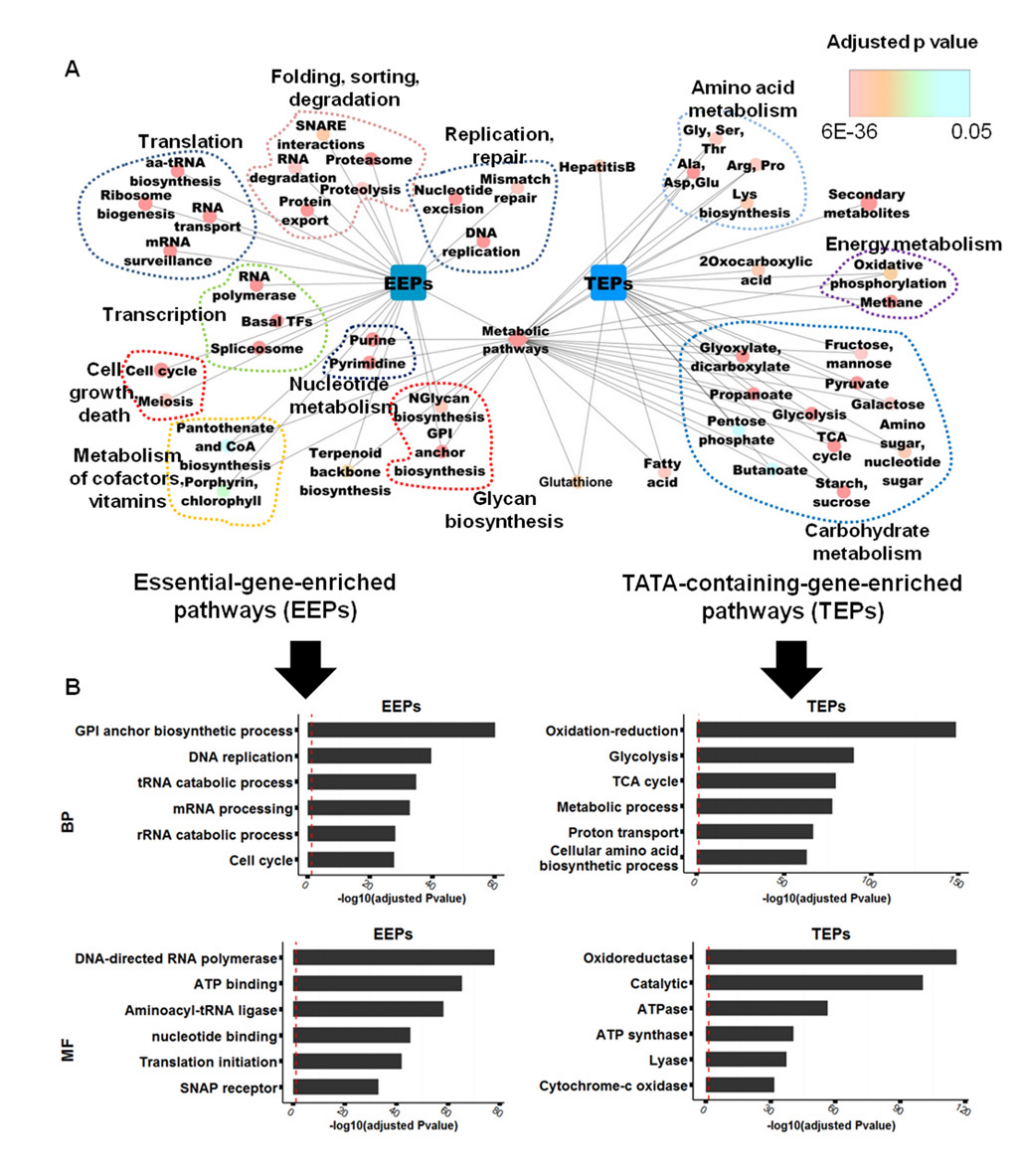
Classification of KEGG pathways into essential-gene-enriched pathways (EEPs) and TATA-containing-gene-enriched pathways (TEPs). (a) We created a network to show which KEGG pathway (ellipse node) belongs to EEPs or TEPs (round rectangle nodes) and which one is included in metabolic pathways (diamond node in the center). The relations are described as edges between two nodes; for instance, glycolysis belongs to TEPs and metabolic pathways. Color (upper-right) represents an adjusted p-value in enrichment analysis. (b) The bar graph shows the results of GO (gene ontology) enrichment analysis in EEPs and TEPs. The red dashed lines correspond to the log transformation of an adjusted p-value of 0.05. BP, biological process; MF, molecular function.

“Metabolic pathways”, which comprises several sub-pathways, was common in both groups of genes (Fig. 1a). However, because “metabolic pathways” is a general term that does not differentiate the two groups, we shifted our analysis to the level of sub-pathways and discarded the term “metabolic pathways” from the list of TEPs and EEPs. Within the metabolic sub-pathways, TEPs and EEPs shared no common elements, indicating that the two groups differ fundamentally with respect to the chemical reactions in which their gene products participate. In gene ontology (GO) enrichment analysis, genes of TEPs were enriched for redox state regulation and metabolic processes, whereas those of EEPs were enriched for replication, transcription, and translation (Fig. 1b).

**Fig 2:**
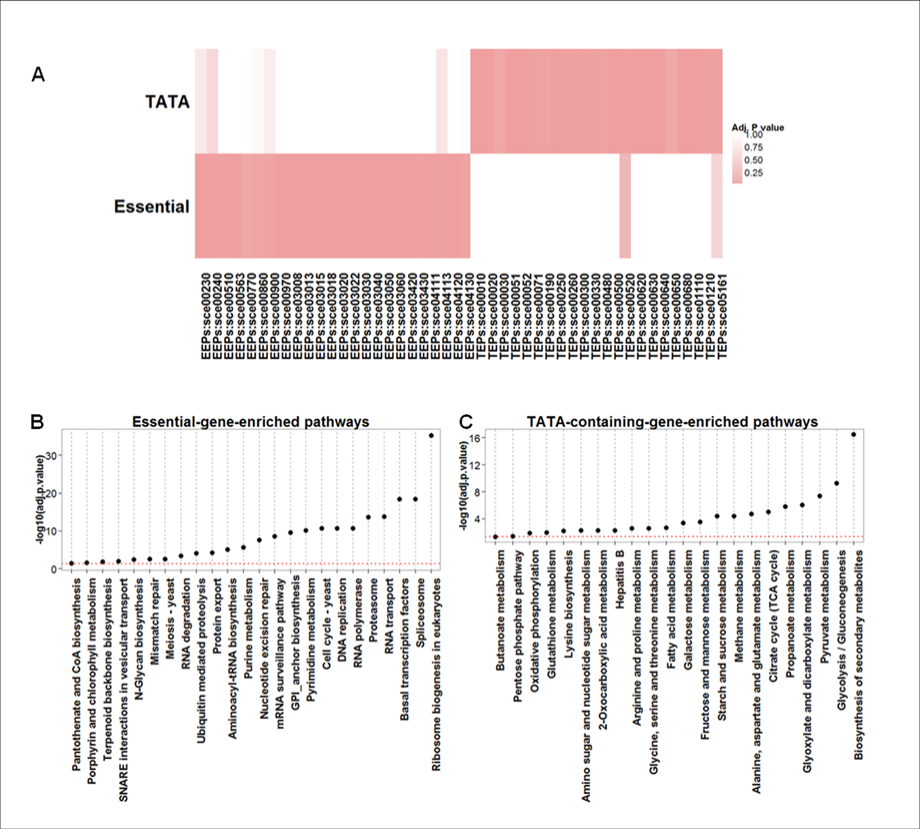
Marked differences in genes and pathways between EEPs and TEPs. (a) The heat map of the adjusted p-values obtained from the enrichment test showed that EEPs are obviously different from TEPs. Rows represent genes and columns pathways (pathway ID); colors represent the negative log transformation of the adjusted p-values, showing the extent to which essential and TATA genes are enriched in the corresponding pathways. (b) Essential-gene-enriched pathways (EEPs) and (c) TATA-containing-gene-enriched pathways (TEPs). The enriched pathways were sorted according to the negative log of the adjusted p-value. The red dashed lines correspond to the log transformation of an adjusted p-value of 0.05.

Most TEPs were associated with metabolism pathways (19 pathways of 22 TEPs), more than half of which were included in the categories of carbohydrate metabolism and amino acid metabolism. In contrast, a large fraction of EEPs were related to genetic information processing, including folding, sorting, degradation, translation, transcription, replication and repair (Fig. 2 a,b and Table 1). Pathways of cell growth and death (2 of 2) were included only in EEPs and were not found in TEPs, indicating that the enrichment of essential or TATA-less genes may direct pathways toward cell survival-related responses (Table 1). Thus, it is conceivable that enrichment of either essential or TATA-containing genes could confer functionally distinct properties on some molecular interactions within the cell, thereby contributing to bipolar regulation.

**Table 1:** KEGG pathway analysis of TATA-less (EEPs) and TATA-containing genes (TEPs).

Group	General description	Functional category^d^	Count (total)^c^
**EEPs** ^**a**^	**Genetic information processing**	**Folding, sorting and degradation**	**5 (7)**
		**Translation**	**4 (5)**
		**Replication and repair**	**3 (6)**
		**Transcription**	**3 (5)**
	**Cell growth and death**	**Cell growth and death**	**2 (2)**
	**Metabolism**	**Glycan biosynthesis and metabolism**	**2 (5)**
		**Metabolism of cofactors and vitamins**	**2 (5)**
		**Nucleotide metabolism**	**2 (2)**
		**Metabolism of terpenoids and polyketides**	**1 (2)**
**TEPs** ^**b**^	**Metabolism**	**Carbohydrate metabolism**	**11 (13)**
		**Amino acid metabolism**	**4 (13)**
		**Energy metabolism**	**2 (4)**
		**Lipid metabolism**	**1 (13)**
		**Metabolism of other amino acids**	**1 (5)**
		**Reaction module maps**	**1 (1)**
	**Human diseases**	**Infectious diseases: Viral**	**1 (1)**

^a^ EEPs—Essential-gene-enriched pathways

^b^ TEPs—TATA-containing-gene-enriched pathways

^c^ The number in parenthesis indicates the number of KEGG pathways involved in the corresponding functional category.

^d^ The metabolic pathways and the biosynthesis of secondary metabolites, which are classified as the global map, were excluded.

### TEPs differ from EEPs at the transcriptional level

**Fig 3:**
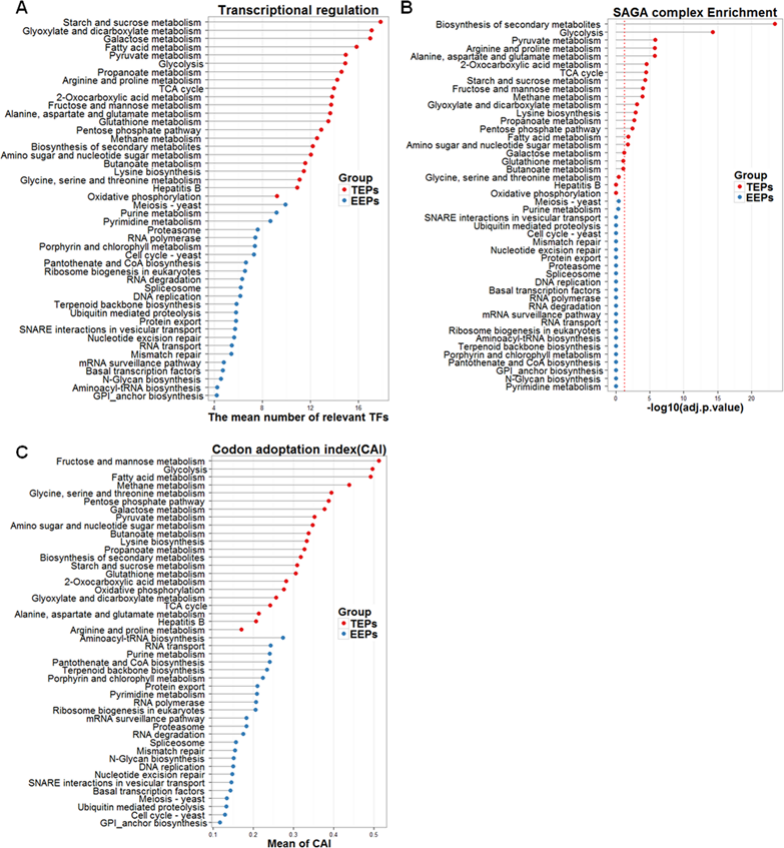
Functional comparison of EEPs and TEPs. (a) Genes of TEPs tend to be associated with a greater number of transcription factors than EEPs. (b) The transcription of TEPs is preferentially associated with the SAGA complex, a regulator of transcription that is known to be related to the stress response. The red dashed lines correspond to the negative log transformation of an adjusted p-value of 0.05. (c) Most TEPs exhibit a higher CAI (codon adaptation index) compared with the EEPs.

The promoters of TATA-containing genes are occupied by regulatory molecules that differ from those bound to TATA-less genes during transcription, resulting in distinct regulatory features, one of which is that TATA-containing genes are regulated more extensively than EEPs at the transcriptional level. Furthermore, the two gene sets show marked preferences for specific coactivators during transcription [2, 3]. Accordingly, we investigated the number of transcription factors and the preferential use of transcription co-activators in TEPs and EEPs (Fig. 3a). Genes of TEPs tend to interact with a greater mean number of relevant transcription factors than genes of EEPs. A statistically significant difference between the two groups in the mean number of relevant transcription factors was found (P < 0.01 in the two-sample t-test).

SAGA and TFIID, which are coactivators of transcription, are responsible for balancing stress- and growth-related cellular responses [3]. Genes that primarily use the SAGA complex during transcription are inclined to be induced and highly regulated by stress. The SAGA complex, therefore, may provide an environment that is conductive to adaptation to stochastic changes within the cell. TFIID is generally associated with growth-related expression or stress-repressed genes. In our analysis, SAGA-dominated genes tended to be enriched in TEPs, whereas EEPs did not exhibit a preference for any coactivators, indicating that TEPs are likely to be stress-related pathways (Fig. 3b).

Indeed, it is reasonable to suggest that the degree of gene expression relies on the specific transcription strategy used to regulate a gene. The extent to which a gene is expressed can be estimated by the codon adaptation index (CAI), a measure that shows the codon preference for a gene during translation. Genes expressed at high levels tend to have greater CAI, meaning that certain codons are used at high frequency for translation [13]. In our results, the indexes of genes in TEPs were significantly higher than those of EEPs (P < 0.01 in the Welch two-sample t-test) (Fig. 3c). Thus, it is proposed that the components of TEPs usually act on each other by maintaining high levels of gene expression. This represents yet another distinction between TEPs and EEPs at the transcriptional level.

### Genes of EEPs have more degrees of interaction and are enriched in more densely connected sub-networks than genes of TEPs

**Fig 4:**
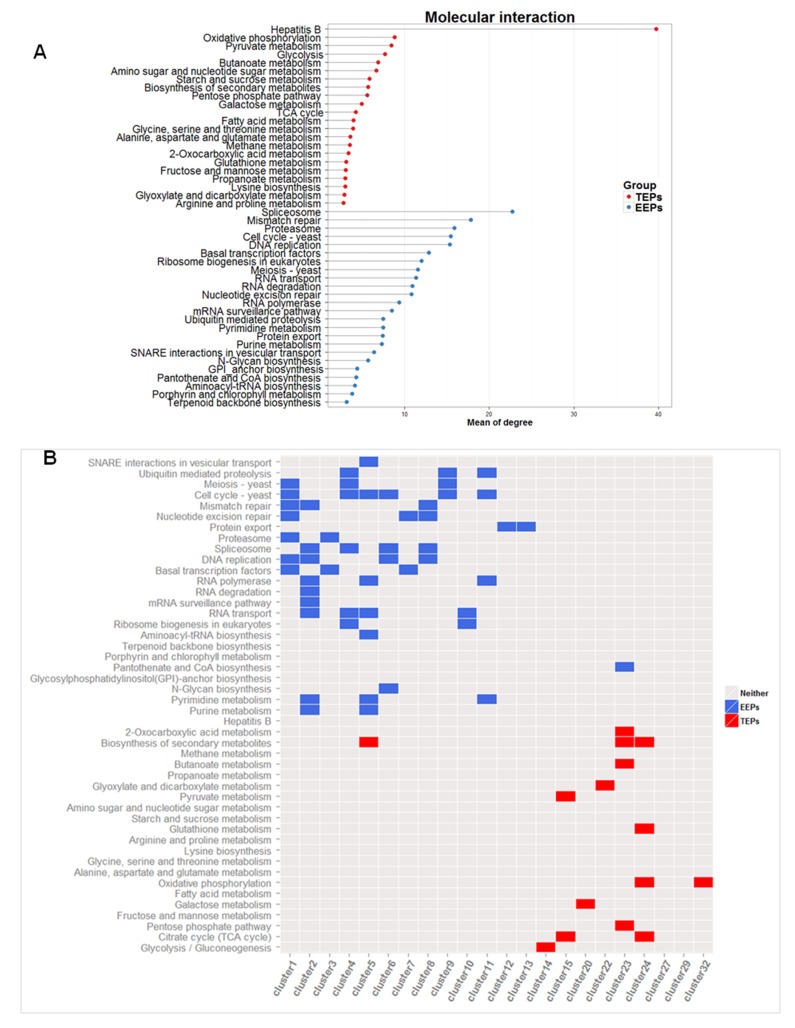
Degrees and connectivity in the interaction network. (a) The mean degrees of molecular interactions are significantly higher (p<0.05) in genes of EEPs than in genes of TEPs. (b) Genes of EEPs were over-represented for higher-scored or highly interconnected clusters, whereas those of TEPs were over-represented for less-scored clusters. The X-axis represents clusters that are displayed in the order of the scores, with the cluster in the left corner having the highest scores.

Cells exploit the strategy of robust expression for maintaining basic functions under conditions of stochastic change. Genes whose protein products perform these basic functions tend to be hubs or essential in the molecular interaction network and to be involved in protein-protein interactions or to have numerous interacting partners [14]. Genes of EEPs are significantly higher in degree (that is, they show a greater number of interactions with other gene products) than genes of TEPs (Fig. 4a, P < 0.05 in the two-sample t-test).

To confirm the network properties of the two groups of genes, we used MCODE analysis to separate the molecular interaction network of yeast into several sub-networks based on the extent of interconnectivity between individual genes. In this analysis, each gene cluster is assigned a score: more densely connected clusters correspond to higher scores, and vice versa. Genes of EEPs were enriched for clusters with higher scores, whereas genes of TEPs were enriched in clusters with lower scores, corroborating that genes with similar interaction properties are likely to cooperate with each other in specific pathways (Fig. 4b). In network theory, highly connected proteins are more essential than less-connected proteins [14]. Our observations thus suggest that EEPs are mainly associated with essential functions and that TEPs are associated with less essential functions.

### Comparison of the metabolic pathways and enzymes in TEPs and EEPs

**Fig 5:**
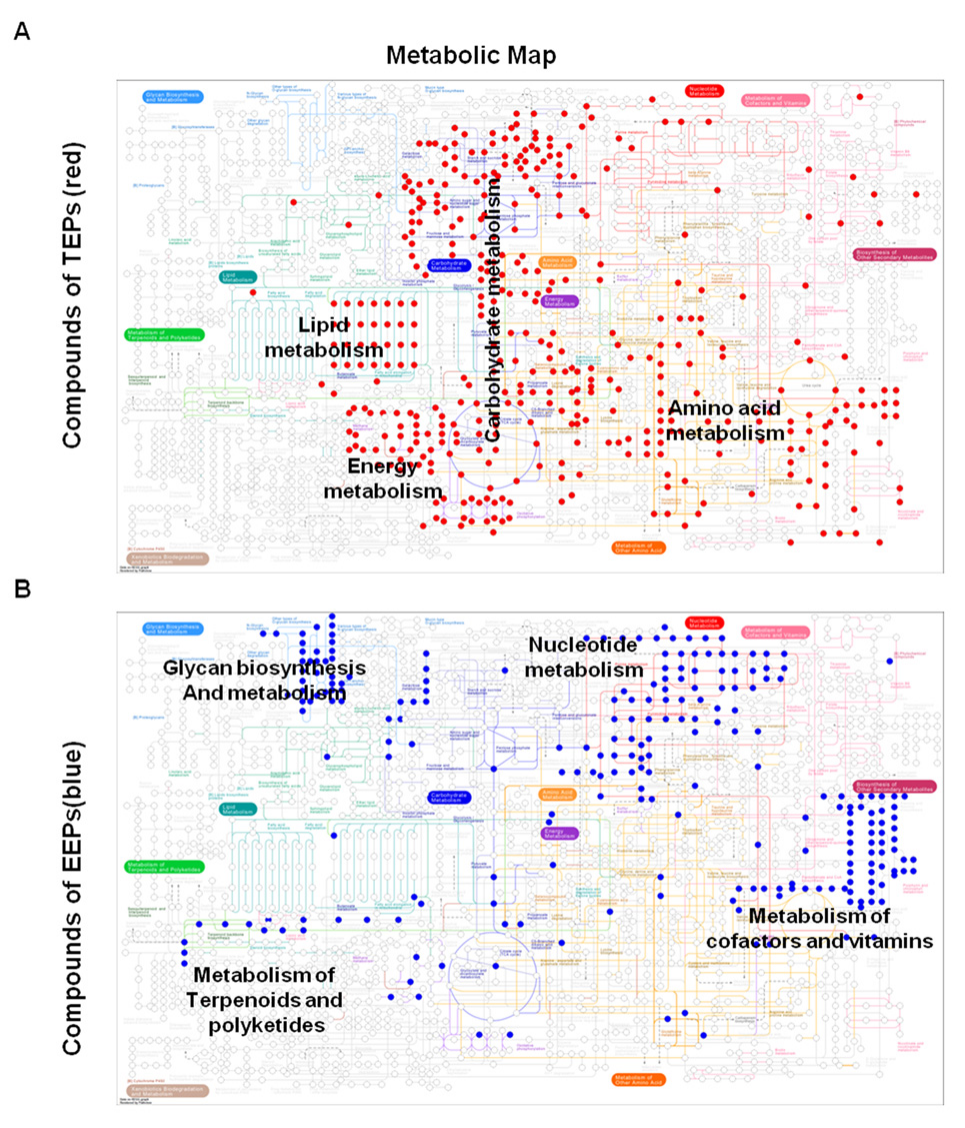
Comparison of pathways in TEPs and EEPs on metabolic map. (a) TEPs and (b) EEPs are associated with different modules in the metabolic pathways analysis; these are highlighted in color (red for TEPs and blue for EEPs). Most of the TEPs in the metabolic pathways are associated with carbohydrate metabolism. Circles indicate compounds in the metabolic pathways.

TEPs and EEPs share no common metabolic pathways, illustrating the completely different roles of these two sets of genes. A vast majority of TEPs (11 of 19) are associated with carbohydrate metabolism. The most intriguing finding is that 11 of the 13 pathways associated with carbohydrate metabolism in KEGG were enriched for TATA-containing genes (Fig. 5a and Table 2). In contrast, EEPs include a different spectrum of metabolic pathways than TEPs, being associated with glycan biosynthesis and metabolism, metabolism of cofactors and vitamins, nucleotide metabolism, and metabolism of terpenoids and polyketides (Fig. 5b and Table 2).

**Table 2:** List of metabolic pathways in TEPs and EEPs.

Group	KEGG pathway	Functional category
**TEP**	**Glycine, serine and threonine metabolism**	**Amino acid metabolism**
	**Alanine, aspartate and glutamate metabolism**	**Amino acid metabolism**
	**Lysine biosynthesis**	**Amino acid metabolism**
	**Arginine and proline metabolism**	**Amino acid metabolism**
	**Pentose phosphate pathway**	**Carbohydrate metabolism**
	**Galactose metabolism**	**Carbohydrate metabolism**
	**Fructose and mannose metabolism**	**Carbohydrate metabolism**
	**Propanoate metabolism**	**Carbohydrate metabolism**
	**Butanoate metabolism**	**Carbohydrate metabolism**
	**Pyruvate metabolism**	**Carbohydrate metabolism**
	**Glycolysis / Gluconeogenesis**	**Carbohydrate metabolism**
	**Glyoxylate and dicarboxylate metabolism**	**Carbohydrate metabolism**
	**Citrate cycle (TCA cycle)**	**Carbohydrate metabolism**
	**Amino sugar and nucleotide sugar metabolisms**	**Carbohydrate metabolism**
	**Starch and sucrose metabolisms**	**Carbohydrate metabolism**
	**Methane metabolism**	**Energy metabolism**
	**Oxidative phosphorylation**	**Energy metabolism**
	**Fatty acid metabolism**	**Lipid metabolism**
	**Glutathione metabolism**	**Metabolism of other amino acids**
	**2-Oxocarboxylic acid metabolism**	**Reaction module maps**
**EEP**	**Glycosylphosphatidylinositol (GPI)-anchor biosynthesis**	**Glycan biosynthesis and metabolism**
	**N-Glycan biosynthesis**	**Glycan biosynthesis and metabolism**
	**Porphyrin and chlorophyll metabolism**	**Metabolism of cofactors and vitamins**
	**Pantothenate and CoA biosynthesis**	**Metabolism of cofactors and vitamins**
	**Terpenoid backbone biosynthesis**	**Metabolism of terpenoids and polyketides**
	**Pyrimidine metabolism**	**Nucleotide metabolism**
	**Purine metabolism**	**Nucleotide metabolism**

**Fig 6:**
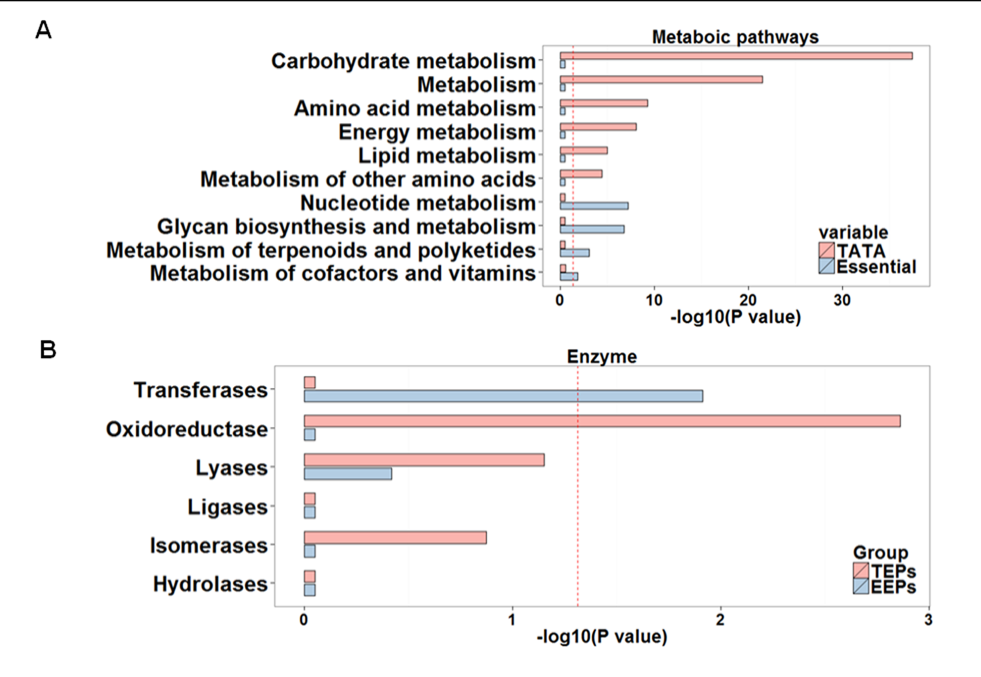
Enrichment of metabolic pathways and enzyme. (a) There is a marked difference in the enriched metabolic pathways between TATA and essential genes. (b) Oxidoreductases are enriched in the enzyme set (197 enzymes) of the TEPs, whereas transferases are enriched in the enzyme set (124) of the EEPs. The red dashed lines correspond to the negative log transformation of an adjusted p-value of 0.05.

The marked difference in the enriched metabolic pathways with which the two sets of genes are associated (Fig. 6a) raises the question of whether the genes in the two groups encode specific enzymes. To facilitate the categorization of enzymatic reactions, we divided the enzymes encoded by genes of TEPs and EEPs into six classes that describe their general functions: oxidoreductases, transferases, hydrolases, lyases, isomerases and ligases. As expected, oxidoreductases catalyzing the transfer of electrons were enriched in the enzyme set of TEPs (P < 0.01), whereas transferases were enriched in EEPs (P < 0.05) (Fig. 6b).

### Regulatory strategies for glycolysis and ribosome biogenesis differ substantially from each other

**Fig 7:**
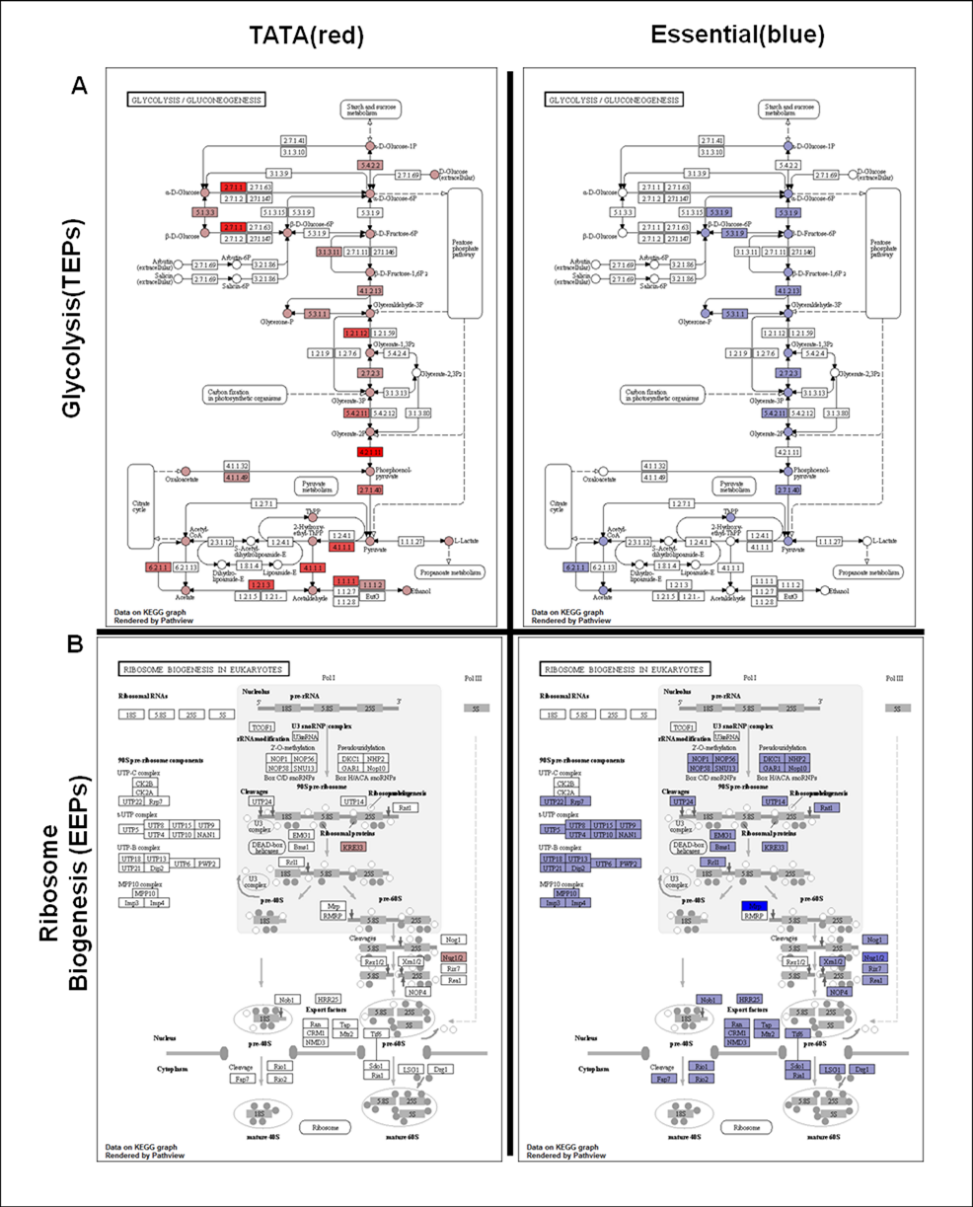
Representative pathways in TEPs and EEPs. (a) Glycolysis is the top-ranked pathway of TEPs. (b) Ribosome biogenesis is the top-ranked pathway of EEPs. Nodes that are highlighted in blue contain at least one essential gene; nodes highlighted in red contain at least one TATA-containing gene. Color intensity is proportional to the number of corresponding genes.

The most enriched and representative pathways in EEPs and TEPs are glycolysis and ribosome biogenesis, respectively (Fig. 7a, b). This indicates the fundamental difference between TATA-containing and TATA-less genes in the sense that glycolysis, the degradation of glucose, is an energy-releasing pathway, whereas ribosome biogenesis, the production of ribosomal protein, is an energy-consuming process. As with other results, it is suggested that to some extent bipolar regulation can be established even in KEGG pathways by the presence or absence of a TATA box element in the promoter region.

## Discussion

Cells are constantly exposed to arbitrary and unpredictable changes in the environment. Failure to cope with these inevitable stimuli can lead to devastating effects on cells, such as disease. For survival, cells usually exploit a bipolar strategy at transcriptional level, using growth-related and stress-related gene expression programs that are mutually exclusive.

Stress-related genes are enriched for TATA boxes in their promoter regions, whereas growth-related genes often display TATA-less promoter elements. In other words, TATA-containing genes tend to be induced by stress, and their expression is typically depressed during the growth-related responses in which TATA-less genes tend to be enriched. However, in the case of certain biological responses, gene products or molecules comprise sets or functional modules rather than undergoing regulation as individual genes or gene products. Our previous genome-wide study in yeast showed that essentiality and the TATA motif in genes are important factors that contribute to cellular housekeeping functions and stress-related responses. The four gene sets classified by the two properties revealed their distinct functions, emphasizing the completely different roles of TATA-containing and TATA-less genes in the context of growth- and stress-related responses. Accordingly, it is reasonable to ask whether the functional outcomes of known molecular interactions such as the KEGG pathway are also affected by the enrichment of TATA-containing or TATA-less genes. In a search of biological networks published online, we used the yeast KEGG pathway as a model of molecular interactions in our study and investigated its properties based on information regarding the presence or absence of TATA box elements in the components of the pathway.

Initially, we divided the KEGG pathway into two groups, TEPs and EEPs, according to the enrichment of TATA-containing or TATA-less genes. The two sets of pathways had no pathways in common. TEPs are mainly related to metabolism, whereas EEPs are usually involved in basic functions of cells such as translation, transcription, replication and repair, and protein modification. For example, the pathway that exhibits the highest enrichment for TATA-less genes is ribosome biogenesis, which contributes substantially to cell growth and proliferation; TATA-containing genes are overrepresented in glycolysis, which is required for converting glucose into pyruvate, which is used for subsequent oxidative phosphorylation generating large amounts of ATP.

A comparison of the number of transcription factors associated with genes in the two types of pathways revealed that TEPs are likely to be extensively regulated at the transcriptional level, a finding consistent with the notion that high levels of intrinsic noise are highly characteristic of stress-related genes with TATA elements [1]. These regulatory properties of TEPs may be suitable for mounting rapid responses to fluctuating environmental conditions. As with individual TATA-less genes, EEPs may tend to require steady-state expression, given that the genes of this set are less stringently regulated during transcription than TEPs.

We also found evidence for two opposite properties of TEPs and EEPs with respect to molecular interactions based on network theory. As expected, the genes of EEPs have more interaction partners than those of TEPs. In MCDOE [9] analysis for finding highly interconnected clusters, the genes of EEPs tend to be over-represented for highly connected clusters, whereas those of TEPs are over-represented for less connected clusters. Like the putative individual roles of TATA-containing and TATA-less genes, these results suggest the robustness of EEPs to perturbation and the variable expression of TEPs.

TFIID and SAGA complexes regulate transcriptional activity differently in the context of cellular stress. SAGA co-activators have been shown to be involved in controlling the expression of stress-induced and highly regulated genes [3]. Our study consistently demonstrated that the SAGA complex is over-represented in most TEPs but not in EEPs, implying that TEPs are highly regulated and that TEPs pathways favor the use of SAGA co-activators for immediate adaptation to rapidly changing environments. Because TFIID is known to regulate approximately 90% of all genes, it is possible that the complex may not be enriched in either group. For analysis of codon preference for translation, genes of TEPs tend to have a higher codon adaptation index than genes of EEPs, suggesting that TEPs may be associated with a higher level of expression than EEPs [15]. If genes of EEPs, similar to those of TEPs, were expressed with high noise, the basic functions of cells would be impaired.

Pathways involved in carbohydrate metabolism were found to be heavily biased toward TEPs (11 of 13 pathways). This makes sense because disruptions in carbohydrate metabolism, such as aberrant regulation of glucose production and insulin resistance, are involved in stress responses [16]. Many genes whose products function in carbohydrate metabolism are induced under environmental stress conditions [17]. At the genome level, TATA box structure contributes to cellular responses to stress [1], and changes or mutations in the sequence-specific elements of TATA boxes could induce a variety of diseases, including metabolic disease [18].

TEPs and EEPs share no common components in metabolic pathways toward which TEPs are biased and show a clear distinction in functional categorization. One EEP is glycan biosynthesis and metabolism; N-linked glycans are related to protein folding, sorting, and degradation [19]. This finding is in agreement with the result that five pathways classified as folding, sorting, and degradation were included in EEPs. Additionally, nucleotide metabolism, an EEP, plays a fundamental role in supplying the building blocks for basic cellular functions. Dysfunctions in purine and pyrimidine metabolism are known to be associated with severe cases of neurological disorders that may occur due to mutations of essential genes in nucleotide metabolism [20]. This result indicates that enrichment of essential genes or TATA-less genes in gene sets may endow pathways with basic function-related processes.

Enzymes classified as oxidoreductases, which are stress-related proteins, are over-represented in TEPs, confirming that TEPs are involved in the stress response and that they thus require variable expression [21]. In genes of EEPs, enzymes classified as transferases are enriched. The primary function of transferases is to alter protein activity by transferring functional groups to the proteins; for example, phophotransferases are responsible for the transfer of a phosphate group from one substrate to another. This finding is consistent with our previous study, in which it was shown that oxidoreductase activity (GOID:16772) is over-represented in TATA-containing genes and that transferase activity (GOID:16491) is over-represented in essential genes [6]. The disparity between EEPs and TEPs can be attributed to the clearly distinguishable strategies used to regulate genes mainly used in each of the two sets: EEPs could be induced under conditions that require drastic changes in the cell's activity to assemble DNA, RNA, and protein and to control housekeeping functions, in which case transferases are likely to be appropriate, whereas TEPs demand flexible responses for adaptation to stress.

A previous study of epigenetic regulation suggested that the observed bipolar regulation can be explained by chromatin stability; that is, stress-induced genes tend to be expressed with a high noise level due to incomplete epigenetic silencing of chromatin. In contrast, complete silencing could be associated with the robust expression of essential genes [22]. In addition to specific promoter elements, differences in the epigenetic state are likely to influence the regulatory strategies used by the cell to control gene expression.

The bipolar regulatory strategies used by cells are likely to influence the cooperative behavior of molecular interactions in KEGG pathways, especially with respect to the cell’s adaptation to the environment. In KEGG pathways, gene products associated with similar strategies (growth-related or stress-related) tend to interact with each other more frequently than those with distinct regulatory features. Thus, two opposite gene properties, namely essentiality and the presence of a TATA box element, can be used to predict the characteristics of gene sets of interest and ultimately to better understand the associated molecular interactions of these gene sets and their contextual properties.

## References

[1] Lopez-Maury L, Marguerat S, Bahler J. Tuning gene expression to changing environments: from rapid responses to evolutionary adaptation. Nat Rev Genet. 2008;9(8):583–93. 10.1038/nrg2398 18591982

[2] Basehoar AD, Zanton SJ, Pugh BF. Identification and distinct regulation of yeast TATA box-containing genes. Cell. 2004;116(5):699–709. 15006352 10.1016/s0092-8674(04)00205-3

[3] Huisinga KL, Pugh BF. A genome-wide housekeeping role for TFIID and a highly regulated stress-related role for SAGA in Saccharomyces cerevisiae. Mol Cell. 2004;13(4):573–85. 14992726 10.1016/s1097-2765(04)00087-5

[4] Yang C, Bolotin E, Jiang T, Sladek FM, Martinez E. Prevalence of the initiator over the TATA box in human and yeast genes and identification of DNA motifs enriched in human TATA-less core promoters. Gene. 2007;389(1):52–65. Epub 2006/11/25. 10.1016/j.gene.2006.09.029 17123746 PMC1955227

[5] Acar M, Mettetal JT, van Oudenaarden A. Stochastic switching as a survival strategy in fluctuating environments. Nat Genet. 2008;40(4):471–5. 10.1038/ng.110 18362885

[6] Han HW, Bae SH, Jung YH, Moon J. Genome-wide characterization of the relationship between essential and TATA-containing genes. FEBS Lett. 2013;587(5):444–51. 10.1016/j.febslet.2012.12.030 23337875

[7] Kanehisa M, Goto S, Kawashima S, Okuno Y, Hattori M. The KEGG resource for deciphering the genome. Nucleic Acids Res. 2004;32(Database issue):D277–80. 14681412 10.1093/nar/gkh063 PMC308797

[8] Luo W, Brouwer C. Pathview: an R/Bioconductor package for pathway-based data integration and visualization. Bioinformatics. 2013;29(14):1830–1. 10.1093/bioinformatics/btt285 23740750 PMC3702256

[9] Bader GD, Hogue CW. An automated method for finding molecular complexes in large protein interaction networks. BMC bioinformatics. 2003;4:2 12525261 10.1186/1471-2105-4-2 PMC149346

[10] Wickham H. ggplot2: elegant graphics for data analysis: Springer; 2009.

[11] Shannon P, Markiel A, Ozier O, Baliga NS, Wang JT, Ramage D, et al Cytoscape: a software environment for integrated models of biomolecular interaction networks. Genome Res. 2003;13(11):2498–504. 14597658 10.1101/gr.1239303 PMC403769

[12] Rivals I, Personnaz L, Taing L, Potier MC. Enrichment or depletion of a GO category within a class of genes: which test? Bioinformatics. 2007;23(4):401–7. 17182697 10.1093/bioinformatics/btl633

[13] Ermolaeva MD. Synonymous codon usage in bacteria. Curr Issues Mol Biol. 2001;3(4):91–7. 11719972

[14] Jeong H, Mason SP, Barabasi AL, Oltvai ZN. Lethality and centrality in protein networks. Nature. 2001;411(6833):41–2. 11333967 10.1038/35075138

[15] Wu G, Culley DE, Zhang W. Predicted highly expressed genes in the genomes of Streptomyces coelicolor and Streptomyces avermitilis and the implications for their metabolism. Microbiology. 2005;151(Pt 7):2175–87. 16000708 10.1099/mic.0.27833-0

[16] Mizock BA. Alterations in carbohydrate metabolism during stress: a review of the literature. Am J Med. 1995;98(1):75–84. 7825623 10.1016/S0002-9343(99)80083-7

[17] Gasch AP, Spellman PT, Kao CM, Carmel-Harel O, Eisen MB, Storz G, et al Genomic expression programs in the response of yeast cells to environmental changes. Mol Biol Cell. 2000;11(12):4241–57. 11102521 10.1091/mbc.11.12.4241 PMC15070

[18] Savinkova LK, Ponomarenko MP, Ponomarenko PM, Drachkova IA, Lysova MV, Arshinova TV, et al TATA box polymorphisms in human gene promoters and associated hereditary pathologies. Biochemistry (Mosc). 2009;74(2):117–29. 19267666 10.1134/s0006297909020011

[19] Helenius A, Aebi M. Roles of N-linked glycans in the endoplasmic reticulum. Annu Rev Biochem. 2004;73:1019–49. 15189166 10.1146/annurev.biochem.73.011303.073752

[20] Micheli V, Camici M, Tozzi MG, Ipata PL, Sestini S, Bertelli M, et al Neurological disorders of purine and pyrimidine metabolism. Curr Top Med Chem. 2011;11(8):923–47. 21401501 10.2174/156802611795347645

[21] Perl A, Hanczko R, Telarico T, Oaks Z, Landas S. Oxidative stress, inflammation and carcinogenesis are controlled through the pentose phosphate pathway by transaldolase. Trends Mol Med. 2011;17(7):395–403. 10.1016/j.molmed.2011.01.014 21376665 PMC3116035

[22] Choi JK, Hwang S, Kim YJ. Stochastic and regulatory role of chromatin silencing in genomic response to environmental changes. PloS One. 2008;3(8):e3002 10.1371/journal.pone.0003002 18714342 PMC2500160

